# Partial Oxidized Arsenene: Emerging Tunable Direct Bandgap Semiconductor

**DOI:** 10.1038/srep24981

**Published:** 2016-04-26

**Authors:** Yu-Jiao Wang, Kai-Ge Zhou, Geliang Yu, Xing Zhong, Hao-Li Zhang

**Affiliations:** 1 School of Chemical Engineering, Nanjing University of Science and Technology, Nanjing, 210094, China; 2 School of Physics and Astronomy, The University of Manchester, Manchester, M13 9PL, UK; 3 College of Chemical Engineering, Zhejiang University of Technology, Hangzhou, 310014, China; 4 College of Chemistry and Chemical Engineering, Lanzhou University, Lanzhou, 730000, China

## Abstract

Arsenene, as a member of the Group V elemental two-dimensional materials appears on the horizon, has shown great prospects. However, its indirect bandgap limits the applications in optoelectronics. In this theoretical work, we reported that partial oxidation can tune the indirect bandgap of arsenene into the direct one. Attributed to the enthalpy decreasing linear to the oxygen ratio, partial oxidized arsenene can be controllably produced by the progressive oxidation under low temperature. Importantly, by increasing the oxygen content from 1O/18As to 18O/18As, the oxidation can narrow the direct bandgap of oxidized arsenene from 1.29 to 0.02 eV. The bandgap of partial oxidized arsenene is proportional to the oxygen content. Consequently, the partial oxidized arsenene with tunable direct bandgap has great potentials in the high efficient infra light emitter and photo-voltaic devices.

Following the great adventure of graphene^1^, elemental two-dimensional (2D) materials have recently attracted extensive attention^2^. Inspired by black phosphorus^3^, people have realized the possible existence of the 2D materials formed by the group V elements. Different from the black phosphorus, arsenene is a monolayer constructed by the arsenic with a space group of R3m^4^, showing a ‘chair’^2^ configuration (Fig. 1a,b). Consequently, arsenene behaves as a semiconductor with indirect bandgap according to the recent theoretical investigations^4^,^5^, which limits the applications in optoelectronics, *e.g.* light emitting diode or photovoltaic devices, because the extra phonon momentum are required to assist the transition and hence results in a far lower efficient of energy conversion than the direct-bandgap semiconductors^6^,^7^. People predicted that by stretching arsenene in biaxial directions or applying high gate voltage up to 4.2 V/nm, the indirect bandgap could be tuned to direct^4^,^5^. However, to stretch other 2D materials experimentally, people have tried with a piezoelectric crystal as substrate, but it significantly increases the complexity of the devices^8^. Therefore, it is still essential to develop a simple method to change the band structure of arsenene.

Chemical modifications have been successfully applied to tune the band structure of graphene and other 2D materials in experiments^9^,^10^,^11^. For instance, by partial h ydrogenation (graphane)^12^, fluorination (fluorographene)^13^ or oxidation (graphene oxide)^14^,^15^, the bandgap of graphene can be opened. The aromatic molecules can selectively n- or p-dope graphene by noncovalent interaction^16^. Partial oxidation can also enhance and red-shift the emission light of MoS_2_ monolayer^17^. Similarly, it is possible to tune the bandgap of arsenene by partial chemical modifications. Compared to graphene, the Group V elemental 2D materials are more chemically reactive. Water or oxygen can react with black phosphorus rapidly in sonication^18^ or under light^19^. Consequently, it has potential to experimentally chemically modify the arsenene in a chemical reaction.

In this work, we applied a first principle simulation to study a group of partial oxidized arsenene with different oxygen contents. The reactivity of arsenene was evaluated by the enthalpy changes in the oxidation, and we also discussed the stable chemical structures and the bond type forming in oxidation. The relationship between electronic band structures and the content of oxygen were established. Furthermore, the change of the bandgap was explained by the analysis on their density of states (DOS).

## Results and Discussions

**Figure 1:**
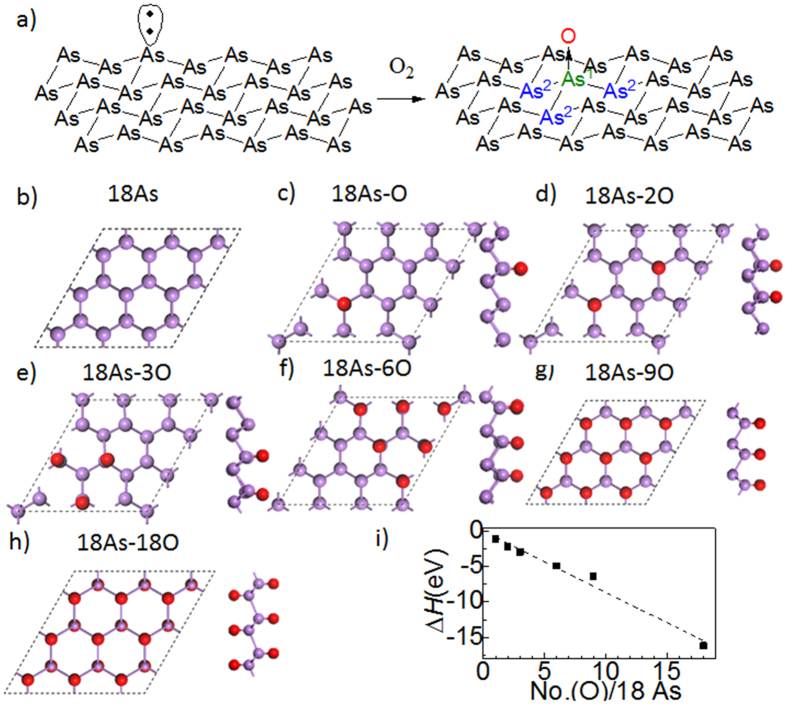
(**a**) The pristine arsenene and one of the oxidized arsenene. We label the arsenic direct linked to oxygen as As^1^, and the arsenic next to As^1^ is As^2^; (**b**) the top view of pristine arsenene. The purple and red ball is the arsenic and oxygen atom, respectively; (**c–h**) the top and cross-sectional view of arsenene oxide (18As-O to 18As-18O). The different models are labelled by the content ratio of As and O. For instance, 18As-O corresponds to a supercell with 18 As and one O; (**i**) the enthalpy of oxidation changes proportional to the oxygen ratio. The dash line is the linear fitting result (R^2^ of 0.990).

In arsenene, the valence electrons are hybridized to three σ-bonds and one lone pair of electrons, forming a buckled monolayer (Fig. 1a,b)^4^. The lone pair of electrons possesses the properties of Lewis’ base, donating the pair into the empty orbitals of other groups. For instance, arsenic can react with air, resulting into their oxides^20^. Therefore, it’s also possible for the arsenene to be oxidized (Fig. 1b). We study a series of partial oxidized arsenenes with different content of oxygen (Fig. 1c–h). The supercell is fixed with18 As, and the oxygen is bonded with the lone pair electron of As. We named the different arsenene oxide as nAs-mO, where n and m is the number of As and O atoms in a supercell, respectively. Generally, all oxygen atoms bond to the sheet in the optimized geometry. Furthermore, we calculate the reaction enthalpy by the following equation:


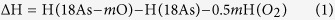


where *m* is the number of oxygen per eighteen arsenics. All enthalpy changes are negative, indicating that the oxidation of arsenene is an exothermic reaction (Fig. 1i). Therefore, the oxidation is to occur even under a low temperature, and would be realized easily. Moreover, the ΔΗ decreases in proportion to the oxygen ratio, and as a result, the partial oxidized arsenene can react with further oxygen. Consequently, one can experimentally tune the input oxygen volume to control the oxygen content in arsenene oxide.

The oxidation changes the crystal structures. We employ the model of 18As-O as an example to show the role of oxygen on the crystal structure. In 18As-O, the bond length between O and closest As^1^ is 1.65 Å, slightly shorter than the As→O length in arsenic acid (ranging from 1.66–1.71 Å)^21^, and the angle of O-As^1^-As^2^ is 119.72°. The in-plane lattice of 18As-O is expanded by 0.25%, however, up to 4% mechanical stretching is able to change the pristine arsenene to a direct band-gap semiconductor^4^. According to the Mulliken charge distribution, the oxygen in 18As-O is negatively charged (0.8 e), indicating that the electron of the arsenic atoms are partially transferred to oxygen, and the chemical bonding between As^1^→O is not ionic. However, the positive charge on As^1^ is lower than 0.8e, suggesting that the charge separation between O and As^1^ also affects the whole arsenene plane. The structure parameters in other oxidized arsenenes are similar to 18As-O (Table S1).

**Figure 2:**
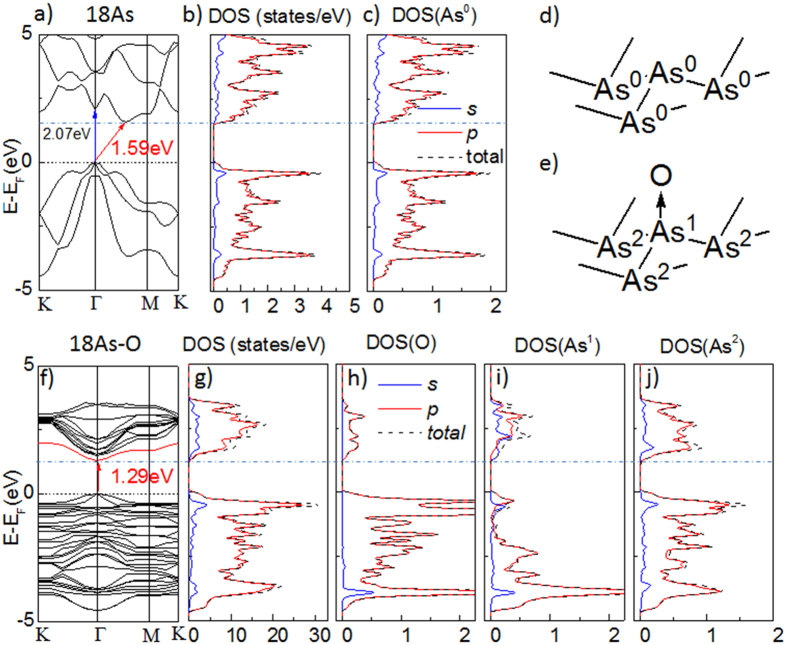
(**a**) The electronic band structure of the pristine 18As. The black dash line indicates the location of Fermi level, and the blue dot-dash line points out the bottom of conductive band (CB); (**b**) the total DOS of the pristine 18As. The dark dash line is the total DOS, while blue and red solid line is the partial DOS contributed by 4*s* and 4*p*-orbitals, respectively; (**c**) the DOS donated by single arsenic atoms are degenerated in pristine arsenene (18As); (**d**) in 18As, where only one type atom exists and named as As^0^. (**e**) in 18As-O where the heteroatomic oxygen was named as O. However, two type arsenic atoms existed: the oxidized and unmodified arsenic were labeled as As^1^ and As^2^, respectively. (**f**) the electronic band structure of the arsenene oxide: 18As-O; (**g**) the total DOS of 18As-O; the DOS donated by of the heteroatom oxygen (**h**), As^1^ (**i**) and As^2^ (**j**), respectively.

Then, we studied the oxidation effect on the electric properties of arsenene. Firstly, we compared the electronic band structure between 18As and 18As-O. The band structure of 18As is similar to the previous works (Fig. 2a)^4^,^5^: the top of the valence band (VB) locate at Γ high-symmetry point, while the bottom of the conduction band (CB) is between Γ and Μ Brillouin zone. Consequently, the pristine 18As has an indirect bandgap of 1.59 eV. In contrast, the direct bandgap transition occurs at Γ to Γ, showing a bandgap of 2.07 eV. The total DOS spectra of pristine 18As are shown in Fig. 2b, and meanwhile, the partial DOS contributed by different orbitals and atoms to the electronic band has also been calculated (Fig. 2c). The 4*p*-electrons of As dominate the electronic states near Fermi level associated with small amount of *s*-electrons, which is consistent with the *sp*^3^ hybridization observed in the previous work^4^,^5^. The contribution of DOS from every arsenic (Fig. 2c) is degenerated and has the feature similar to the total DOS, as all arsenic is the same in terms of chemical environment (Fig. 2d).

However, the modification of oxygen divides the arsenic into two types as shown in Fig. 2e: the oxidized As^1^ and the rest elemental arsenic, e.g., As^2^. Consequently, the oxidation significantly changes the band structure. In 18As-O, the bottom of CB shift to the Γ point, locating at the same Brillion zone of the VB top (Fig. 2f). Meanwhile, the top of the VB remains at the same location, and maintains the similar feature as the pristine arsenene. Therefore, the oxidation makes the bandgap of arsenene direct, and reduces the direct band gap down to 1.29 eV. Besides, the oxidation splits the orbitals of arsenene, increasing the number of CB bands closed to Fermi level, resulting in a significant increase of the total DOS in Fig. 2g. The electronic states of 18As-O are also constructed by *p* and *s*-orbitals. The VB band top of 18As-O also reaches the Fermi level, in contrast, a shoulder peak in the total DOS appears at the bottom edge of CB, which narrows the bandgap consequently. Interestingly, both *p* and *s*-orbitals give contribution to the shoulder peak at the CB bottom edge here.

To understand the origin of the direct bandgap in 18As-O, the partial DOS spectra donated by different atoms are analysed. Compared to the degenerated chemical environment in pristine arsenene, the electronic states from every atom in 18As-O have been significantly changed. For the heteroatom, O is shown in Fig. 2h, where the electronic states near Fermi level is mainly constituted by its *p*-orbitals, particularly in CB region. Meanwhile, the *s*-orbitals of O do not participate in the electronic states near Fermi level. Therefore, O provides the *p*-orbital to receive the electrons from arsenic and form the chemical bond with it. The minimum of CB electronics states from the *p*-orbital of O locates touches 1.29 eV, the bottom of CB (Fig. 2h), therefore, O only provides partial contribution to the bottom band in CB region.

The arsenic atom As^1^, directly linked to O exhibits remarkable difference in its DOS spectra (Fig. 2i) from the pristine arsenic atom in 18As. The minimum peak of its total DOS in the CB region locates at 1.37 eV and extends to 1.21 eV, indicating that part of the bottom band reaching 1.29 eV at Γ point (Fig. 2f) has the contribution from As^1^. Moreover, the main body of the minimum peak in CB region of As^1^ comes from its 4s-orbital, instead of the 4*p*-orbitals. Consequently, the 4*s*-orbital provided by As^1^ is one of the reasons to make the direct bandgap in 18As-O. Compared to the pristine arsenene, the lone pair of electrons from arsenic atom is used to form the bonding with O, so the 4*p*-orbital is tightly bound and reduces the contribution towards the electronic states at the VB top and CB bottom significantly. As a result, the 4*s*-orbital from As^1^ was split to form the new states near Fermi level.

The oxidation influence also spreads to other arsenic atoms. For instance, the DOS of As^2^, the one next to As^1^ has been changed particularly in CB region (Fig. 2j). Its VB region has similar feature as the pristine arsenic in 18As (Fig. 2c). However, a shoulder peak shows at the minimum of CB extended to 1.22 eV, contributed by the 4*p*-orbitals of As^2^, according to its partial DOS. Overall, we can conclude that the new appeared band acting as the bottom of CB is a result of the 4*s*-orbitals of the oxidized arsenic (As^1^) slightly combined with *p*-orbitals of oxygen and unoxidized arsenic (As^2^).

**Figure 3:**
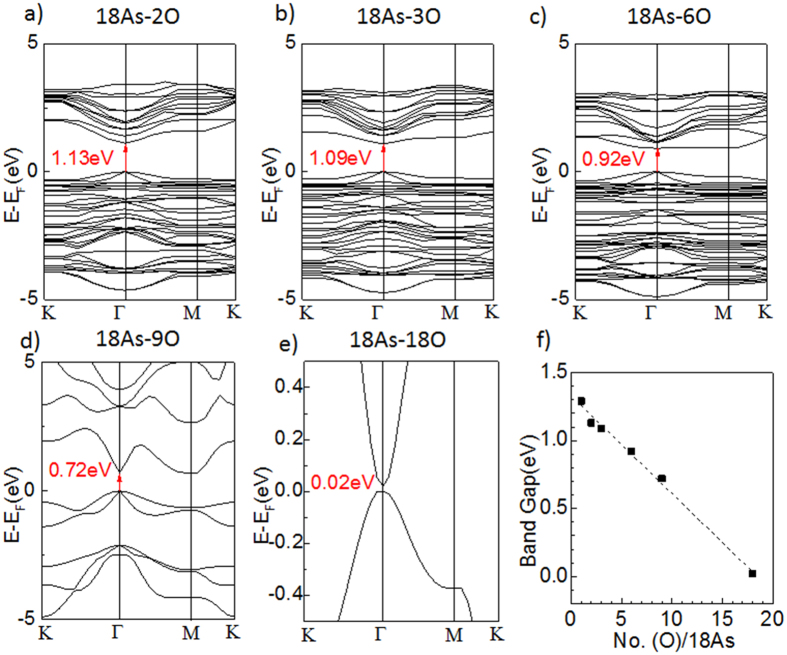
(**a–e**) The electronic band structure of 18As-2O, 18As-3O, 18As-6O, 18As-9O, 18As-18O. (**f**) the relationship between the bandgap and the content ratio of oxygen.

By hydrogenation or fluorination, people can artificially control the yield of chemical modification in order to widen the bandgap of graphene^22^,^23^,^24^. Accordingly, It is also possible to tune the yield of oxidation for arsenene in experiment. Here, we investigate the relation between the bandgap and the oxygen content (Fig. 3). The number of arsenic atoms is fixed to 18, and the number of oxygen is set from 1 to 18, respectively. The configurations of As-O bond in those models are similar to 18As-O (Table S1 in the supporting information), however, the bandgap varies with the content of oxygen (Fig. 3a–e). All five oxidized arsenenes show direct bandgap and are consistent with the 18As-O, because the bottom of their CB move to the Γ point and march the top of VB. In contrast, the top of the VB remains at Γ point, regardless of the oxygen ratio. If all lone pairs of electrons of arsenic atoms are saturated by oxygen (18As-18O), the bandgap will be narrowed down to 0.02 eV. Interestingly, the direct bandgap of arsenene oxide is narrowed with the content of oxygen increased, and the width of the bandgap is reduced in proportion to the content of oxygen (Fig. 3f) as:


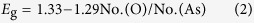


where *E*_g_ is the band gap and No. (O) and No. (As) is the content of oxygen and arsenic, respectively.

Similar to the 18As-1O, the direct bandgap from 18As-2O to 18As-18O can be also understood by analysing their partial DOS (supplementary information, Figure S2–S6). Their top of the CB are constructed by the *s*-orbital of the oxidized arsenic atoms and the *p*-orbital of the oxygen and unoxidized arsenic atoms. Compared to the *p*-orbitals from pristine arsenic near the top of CB, the *p*-orbitals of the oxidized arsenic significantly decrease, because the electrons are withdrawing from the arsenene plane to the oxygen. The electron transfer phenomenon is consistent with the charge separation in Table S1. The orbital locations of CB are independent with the increase of oxygen ratio, corresponding well with the observations on their band structure. For every partial oxidized arsenene, the bottom of VB is mainly constituted by the *s*-orbital of the oxidized arsenic atoms and the *p*-orbital of unoxidized arsenic atoms Meanwhile the oxygen donates little contribution to the bottom of CB. With the increasing oxygen content, the orbitals donated from the arsenene continuously shift down, leading to the observations on their band structure.

It is worthy of comparing the oxidation on the electronic structure between group V and IV 2D materials. Previously, similar chemical modification, e.g., oxidation, fluorination and hydrogenation normally open or widen the bandgap of group IV 2D materials, e.g., graphene^25^, silicene^26^,^27^ or germanene^28^,^29^. Taking graphene as an example, every atom provides an out-of-plane *sp*^2^ hybridized orbital with one single electron. As the electrons tend to appear in pairs, a delocalized conjugation bond is formed over the atomic layers, making graphene a zero-bandgap semiconductor. Once other atoms (hydrogen, oxygen, fluorine etc.) attaches on graphene surface, part of the electrons has to quit from the delocalized conjugated bond in order to link with the chemical group, and consequently create the defect to block the electron flow and open the bandgap. However, every atom in a group V 2D sheet contributes a lone pair of electrons localized, and all orbitals are fully occupied, and consequently the electron flow is not as easy to flow. If the lone pair of electrons is shared with other atoms (e.g., oxygen), an electron-deficient site is created on the plane of 2D sheet, making the electron flow easily and narrowing the bandgap. With more electron-deficient site introduced by the oxidation, the bandgap will be consequently narrowing continuously in group V arsenene.

**Figure 4:**
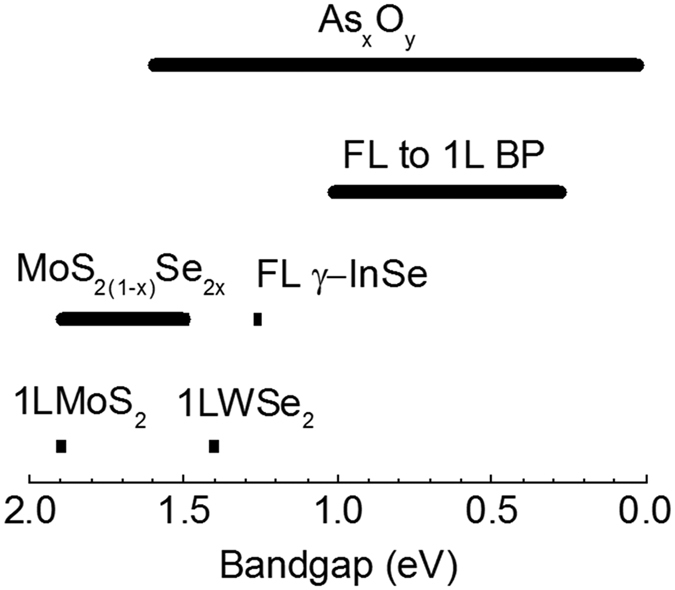
The bandgaps of the partial oxidized arsenene (As_x_O_y_) and existing direct bandgap semiconductors: MoS_2_ monolayer (1L MoS_2_), WSe_2_ monolayer (1L WSe_2_), the 2D alloy of MoS_2_ and MoSe_2_ (MoS_2(1−x)_Se_2x_), few layered γ-InSe (FL γ-InSe) and few to mono-layered black phosphorene (FL to 1L BP).

Moreover, compared to the previous direct bandgap semiconductors, the group of partial oxidized arsenenes exhibits variable bandgaps distributed over a wider range from visible to near-infra region (Fig. 4). Mostly one 2D material shows one specific bandgap. For instance, the mono-layered MoS_2_ and WSe_2_ have a direct bandgap of 1.9^6^ and 1.4 eV^30^, respectively. 20 nm thick γ-InSe shows a bandgap of 1.26 eV^31^. Consequently, it is very difficult for the semiconductor industry to fabricate a group of semiconductors with variable bandgaps by slightly tuning the composition ratio, e.g., Al_x_Ga_(1−x)_As^32^. Recently, by manipulating the Se composition, Zhang and Bartels have successfully tune the bandgap of MoS_2(1−x)_Se_2x_ alloy from 1.5–1.9 eV in visible region, respectively^33^. Moreover, the bangaps of mechanically exfoliated few-layered black phosphorene was found to vary from 0.3 to 1 eV depending on the number of layers^34^. Apparently, it is a great challenge to precisely control the number of layers in Scotch-tap exfoliation. In contrast, the group of partial oxidized arsenene provide a way to make semiconductors with variable bandgaps from visible to near infra region by controlling the element composition, which is familiar to the semiconductor industry.

Here, we would also like to compare our method with mechanical stretching and external electric field^4^,^5^. People has reported that a biaxial in-plane stretching higher than 2% in length would change the pristine arsenene to the direct bandgap^5^. Several experimental methods are available to stretch the 2D materials. For instance, pressing the suspended 2D sheet by AFM^35^ or blowing a balloon^36^ to realize the biaxial stretching over 2%. However, the additional mechanical accessories will make the individual device complicated, limiting the application in the large-scale integrated circuits. People also proposed that in a van der Waals heterostructure, the lattice mismatch could cause 2D sheets stretched^4^, however, the experimental records on the stretching of 2D sheet in van der Waals heterostructures are only 0.3%^37^. Kamal *et al*. proposed that by applying vertical electric field up to 4.2 V/nm to bring direct bandgap, but it is difficult to realize such higher strength of the electric field as they admitted^5^. Compared with above methods, it is easier to realize the oxidation of layered materials. We know that graphene can be oxidized by harsh chemical reagents^38^ and the monolayer of transition metal dichalcogenides can react with ozone^39^. In contrast, a simply aging process can oxidize the arsenic bulk^40^. Our analysis on the change of enthalpy shows that the oxidation is able to occur under low temperature. We believe it would be easy to oxidize the arsenene once it was obtained. Very recently, Materl *et al*. reported a simple photo-oxidation of black phosphorene under the existence of water, from which they have proved that the oxygen content can be tuned by the oxygen gas pressure and the light intensity^19^. We believe that the same technique would be used to approve our simulation soon after the pristine arsenene was synthesized. Besides, we have found that the bandgap of arsenene can be quantitatively tuned from near-infra to infra region by controlling the content of oxygen. Therefore, to make a direct bandgap arsenene, it is more applicable to employ the oxidation than mechanical stretching.

## Conclusions

In conclusion, we have demonstrated that arsenene oxide can transit its bandgap from an indirect to a direct one. The transition is owing to a new CB bottom band composed of the 4*s*-orbital of the oxidized arsenic combining with the *p*-orbitals of oxygen and unoxidized arsenic. The width of the direct bandgap is narrowed from near-infra to infra region in proportional to the oxygen content. Our work has provided a chemical way to make a group of arsenene derivates with direct bandgap, and will inspire its future applications in optoelectronics.

## Computational Methods

All calculations were performed using the plane wave code CASTEP^41^ under the general gradient approximation (GGA) expressed by PBE functional^42^. A 3 × 3 supercell of arsenene was used in all simulation. All of the structure models were fully relaxed, including the lattice cells, until the forces smaller than 0.01 eV/Å and the energy tolerances less than 5 × 10^−6 ^eV per atom. A vacuum of 20 Å between these 2D layers was used with 13 × 13 × 1 Monkhorst–Pack k-points and a plane-wave cutoff energy of 550 eV for the geometry optimization, while the Monkhorst–Pack *k*-points were extended up to 31 × 31 × 1 to give more precise electronic band structures and DOS.

## Additional Information

**How to cite this article**: Wang, Y.-J. *et al*. Partial Oxidized Arsenene: Emerging Tunable Direct Bandgap Semiconductor. *Sci. Rep.* **6**, 24981; doi: 10.1038/srep24981 (2016).

## Supplementary Material

Supplementary Information
