# Supplementary material for: Partial Oxidized Arsenene: Emerging Tunable Direct Bandgap Semiconductor
Source: Sci Rep. 2016 Apr 26;6:24981. doi: 10.1038/srep24981 (PMC4845026; doi:10.1038/srep24981)
Supplement: Supplementary Information [file srep24981-s1.pdf]

Supporting Information

Partial Oxidized Arsenene: Emerging Tunable Direct Bandgap Semiconductor

Yu-Jiao Wang[1], Kai-Ge Zhou[2]*, Geliang Yu[2], Xing Zhong[3], and Hao-Li Zhang[4]

[1] School of Chemical Engineering, Nanjing University of Science and Technology, 210094, China

[2] School of Physics and Astronomy, The University of Manchester, M13 9PL, UK

[3] College of Chemical Engineering, Zhejiang University of Technology, 310014, China

[4] College of Chemistry and Chemical Engineering, Lanzhou University, 730000, China

1. The Structures of Oxidized Arsenenes

1.1 The Configurations of Partial Oxidized Arsenene

Up to now, two pristine arsenene were predicted: *Pmna* and *P*3*m*1 symettric configurations, respectively[1]. In this work, we only discussed *P*3*m*1 configuration arsenene, which is more stable than the other. Meanwhile, since the natural grey arsenene is constructed by stacking of *P*3*m*1 arsenene, we believe the discussions on *P*3*m*1 configuration are more useful for future experimental investigations.

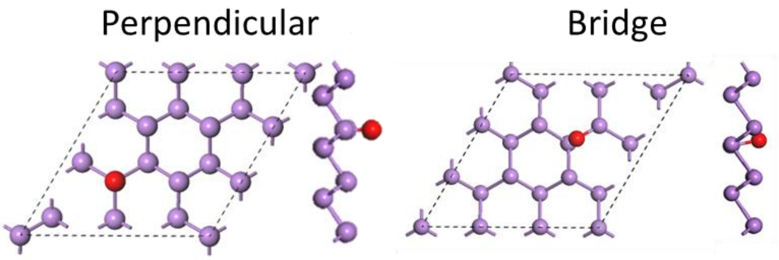

Figure S1 two possible configurations of 18As-O: perpendicular (left) and bridge (right).

The bonding of the oxygen has two possible configurations (Figure S1). The perpendicular configuration is as we discussed in main text. In the other one, oxygen insert into an As-As bond, and make the arsenene plane distorted. As a result, the calculated energy of bridge configuration is 0.07eV larger than the perpendicular one. Moreover, in photo-oxidized black phosphorene, the P_xO_y was found to be similar to phosphoric acid, rather than phosphorous acid, according an experimental XPS result[2]. In phosphoric acid, the P has four bonds, similiar to the perpendicular configurations. In contrast, the P in phosphorous acid has three bonds, similar to the bridge one. Another theoretical work also support their finding[3]. Therefore, we focus on the perpendicular configuration in this work.

1.2 The Structure Parameters of Oxidized Arsenenes

The bond length and angle of As-O bond are shown in table S1 as well as the charge distributions of arsenic and oxygen. Note that in 18As-O, 18As-9O and 18As-18O, only one type of As-O exists, while there are more than one type in other models. Therefore, we give the average value for 18As-2O to 18As-6O.

Table 1 The bonding length, angle, and charge distributions of the oxidized arsenenes.

**Model**	**L(As^1-O)/ Å**	**θ(O-As^1-As^2)/°**	***q*(O)/*e***	***q*(As^1) /*e***
**18As-O**	1.65	119.72	-0.80	0.65
**18As-2O**	1.65±0.00	116.62±0.18	-0.80±0.00	0.65±0.00
**18As-3O**	1.64±0.00	118.39±4.90	-0.77±0.00	0.72±0.00
**18As-6O**	1.64±0.01	120.01±2.44	-0.72±0.00	0.72±0.00
**18As-9O**	1.62	120.85	-0.72	0.72
**18As-18O**	1.63	112.62	-0.71	0.71

2. Band Structure of Oxidized Arsenenes

2.1 18As-2O

The atoms in 18As-2O are diverse (Figure S2a). We label the two oxygen atoms in a lattice cell as O^1 and O^2, respectively, and the oxidized arsenic as As^{n1} (n=1, 2), where n corresponds to the serial number of oxygen, while the arsenic next to As^{n1} is named as As^{n2}.

The bandgap is narrowed down to 1.13eV from the 1.29eV in 18As-O (Figure S2b). However, the features of density of states (DOS) are similar between 18As-O and 18As-2O (Figure S2c-i). The bottom conducting band (CB) mainly from the s-orbital of oxidized arsenic (Figure S1f and h) and partially from p-orbitals of unoxidized arsenic (Figure S2g and i) and oxygen (Figure S2d and e).

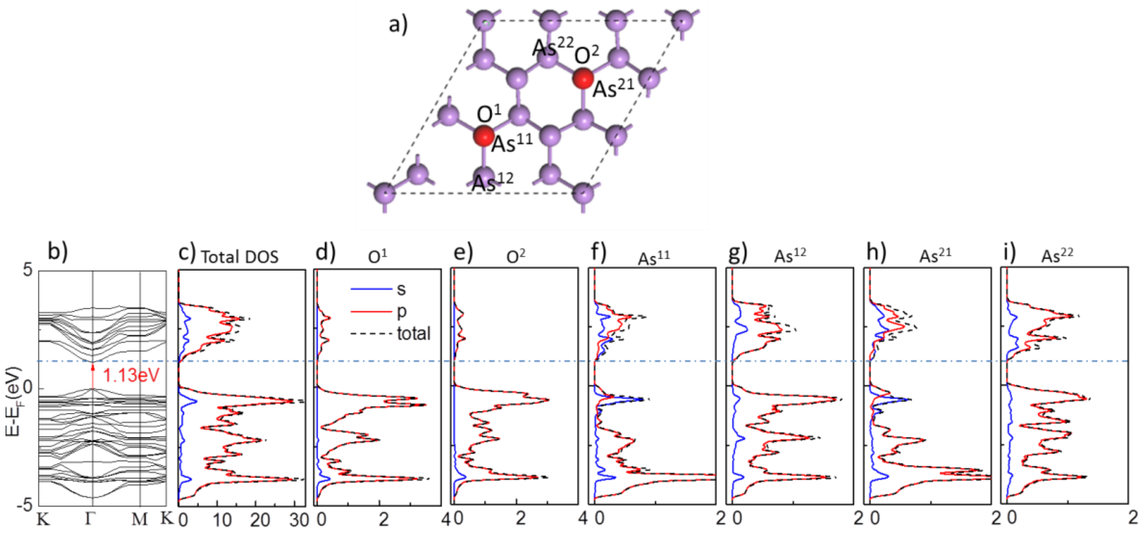

Figure S2 a) the top view of 18As-2O. The arsenic and oxygen atoms are purple and red balls, respectively. The atoms around the As-O bonds are labelled for the following analysis; b) the band structure of 18As-2O; c) total density of states (DOS) of 18As-2O; d) to i) the contribution from the labelled atoms in a) to the DOS.

2.2 18As-3O

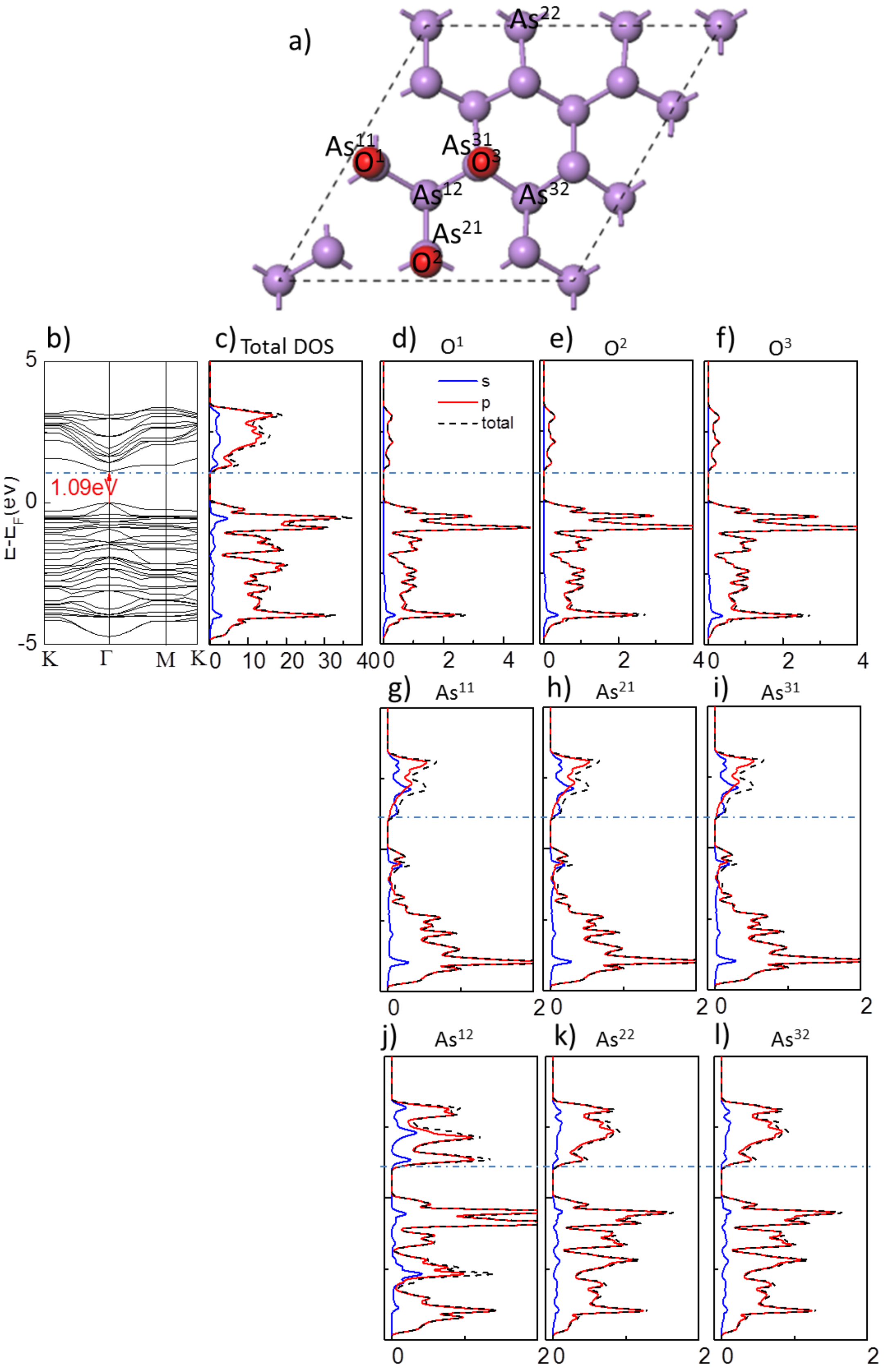

Figure S3 a) the top view of 18As-3O. The arsenic and oxygen atoms are purple and red balls, respectively. The atoms around the As-O bonds are labelled for the

following analysis; b) the band structure of 18As-3O; c) total density of states (DOS) of 18As-2O; d) to l) the contribution from the labelled atoms in a) to the DOS.

In Figure S3a, we label the atoms in 18As-3O in the same way as 18As-2O. Its bandgap is further narrowed down to 1.09eV (Figure S3b). The contribution from *s*-orbials to the CB bottom has signifcantly increased. According to the analysis on each atoms, we find that they are from *s*-orbitals of unoxidized arsenic, particularly the As^{12} between three oxidized arsenic.

2.3 18As-6O

The atoms in 18As-6O are labeled as before. Similar to 18As-3O, we observe the participation of the *s*-orbitals of unoxidized arsenic into the CB bottom.

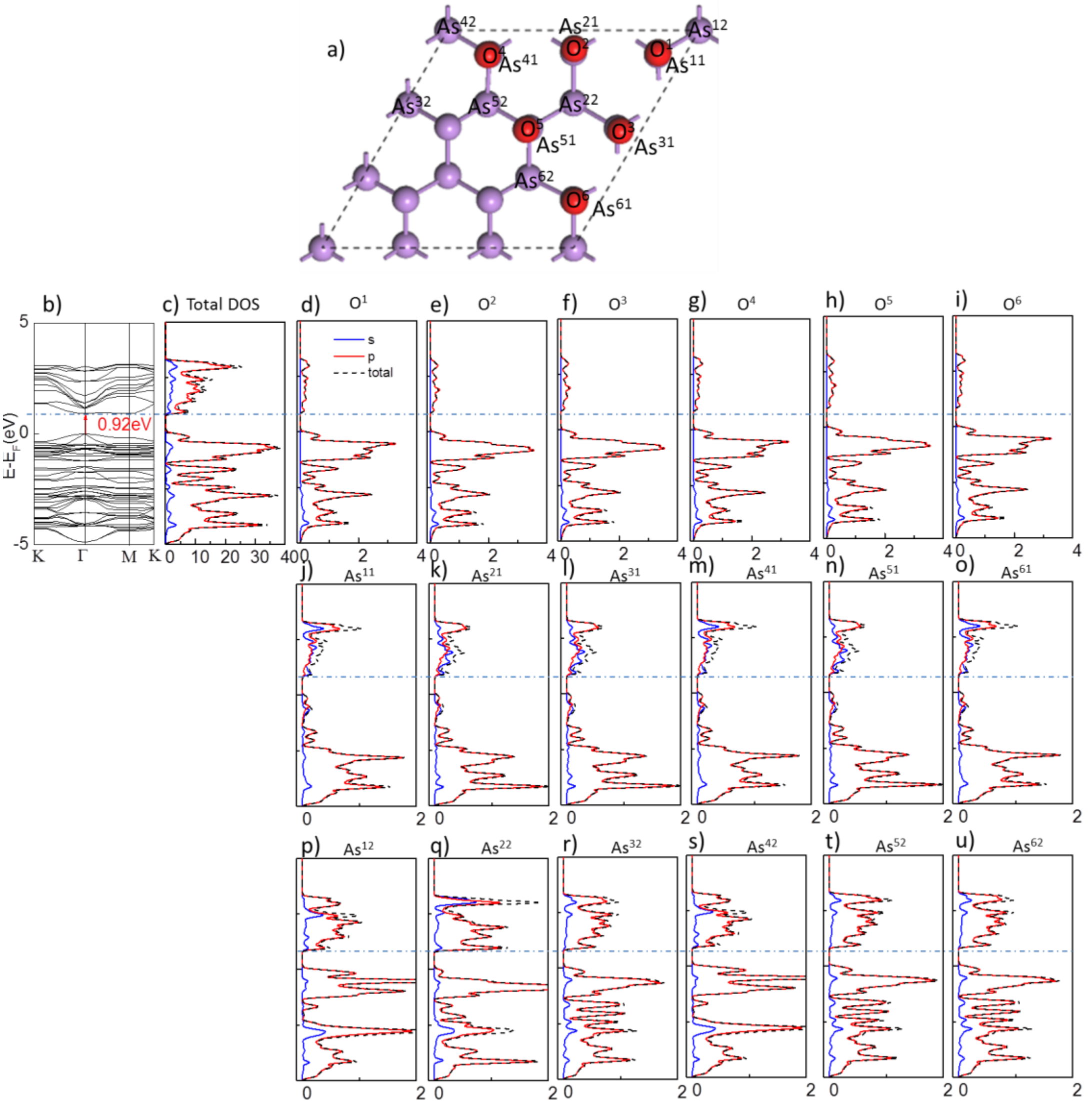

Figure S4 a) the top view of 18As-6O. The arsenic and oxygen atoms are purple and red balls, respectively. The atoms around the As-O bonds are labelled for the following analysis; b) the band structure of 18As-6O; c) total density of states (DOS) of 18As-6O; d) to u) the contribution from the labelled atoms in a) to the DOS.

2.4 18As-9O

In 18As-9O, the atom types have been degenerated due to the lattice symmetry. It has only one oxygen, and two arenic (oxidized and unoxidized) types. The CB bottom are mainly contructed by the *s* and *p*-orbital from oxidized arsenic (As^1), *p*-orbital from unoxidized arsenic (As^2), and partially *p*-orbital from oxygen (O).

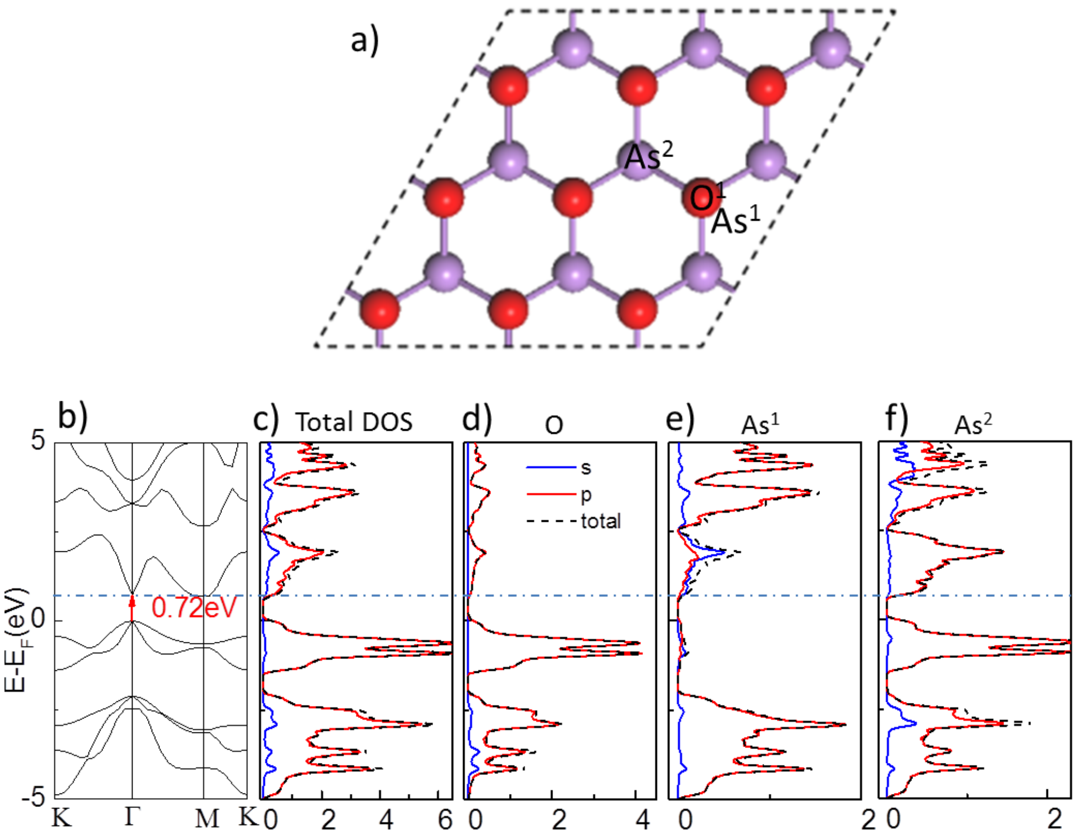

Figure S5 a) the top view of 18As-9O. The arsenic and oxygen atoms are purple and red balls, respectively. The atoms around the As-O bonds are labelled for the following analysis; b) the band structure of 18As-9O; c) total density of states (DOS) of 18As-9O; d) to f) the contribution from the labelled atoms in a) to the DOS.

2.5 18As-18O

In 18As-18O, only one oxygen and one arsenic are left. The CB bottom are only from the *p*-orbital both from oxygen and arsenic.

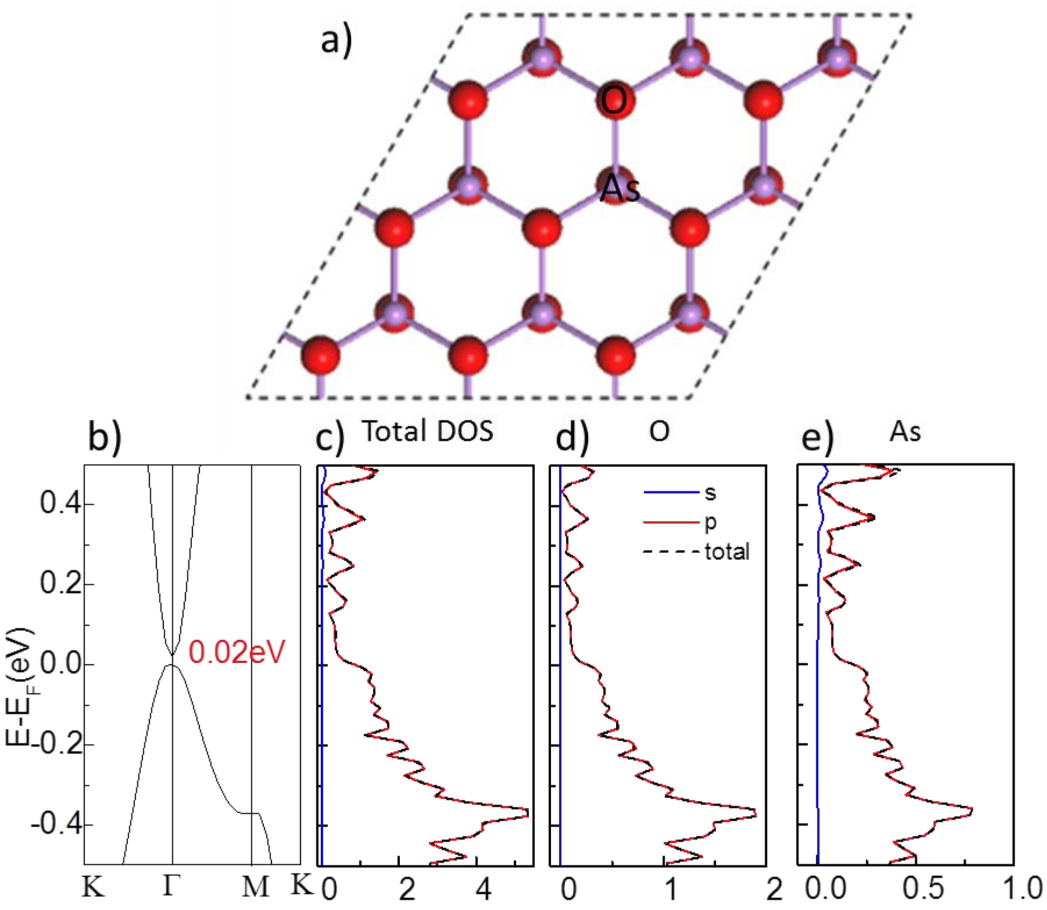

Figure S6 a) the top view of 18As-18O. The arsenic and oxygen atoms are purple and red balls, respectively. The atoms around the As-O bonds are labelled for the following analysis; b) the band structure of 18As-18O; c) total density of states (DOS) of 18As-18O; d) to e) the contribution from the labelled atoms in a) to the DOS.

Reference:

1. Kamal, C. & Ezawa, M. Arsenene: Two-dimensional buckled and puckered honeycomb arsenic systems. *Phys. Rev. B* **91**, 085423 (2015).
2. Favron, A. *et al.* Photooxidation and quantum confinement effects in exfoliated black phosphorus. *Nat Mater* **14**, 826-832 (2015).
3. Wang, G., Pandey, R. & Karna, S. P. Phosphorene oxide: stability and electronic properties of a novel two-dimensional material. *Nanoscale* **7**, 524-531 (2015).
